# Supplementary material for: Modality-specific and modality-general representations of subjective value in frontal cortex
Source: Commun Biol. 2024 Nov 21;7:1550. doi: 10.1038/s42003-024-07253-8 (PMC11582727; doi:10.1038/s42003-024-07253-8)
Supplement: Supplementary file 2 — Supplementary Information [file 42003_2024_7253_MOESM2_ESM.pdf]

Supplementary Information to “Modality-specific and modality-general representations of subjective value in frontal cortex”

Dang et al.

Supplementary Figures

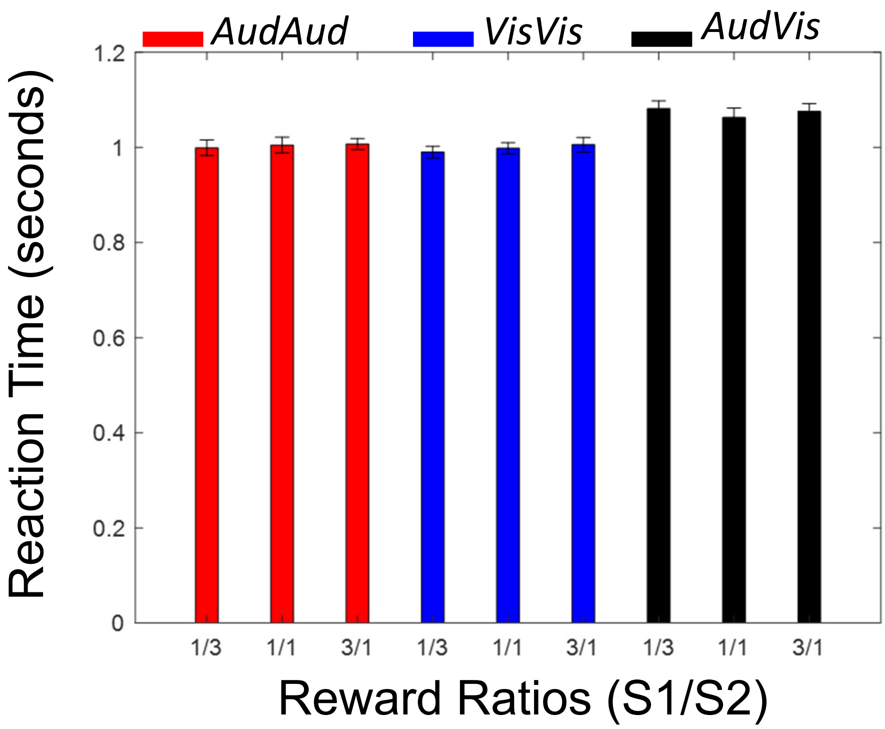

Figure S1. Reaction Times in the value task. Mean RTs across participants for each reward ratio {1:3, 1:1, 3:1} of options $S_1 : S_2$ separately for each modality condition of the value task (*AudAud*, *VisVis* and *AudVis*).

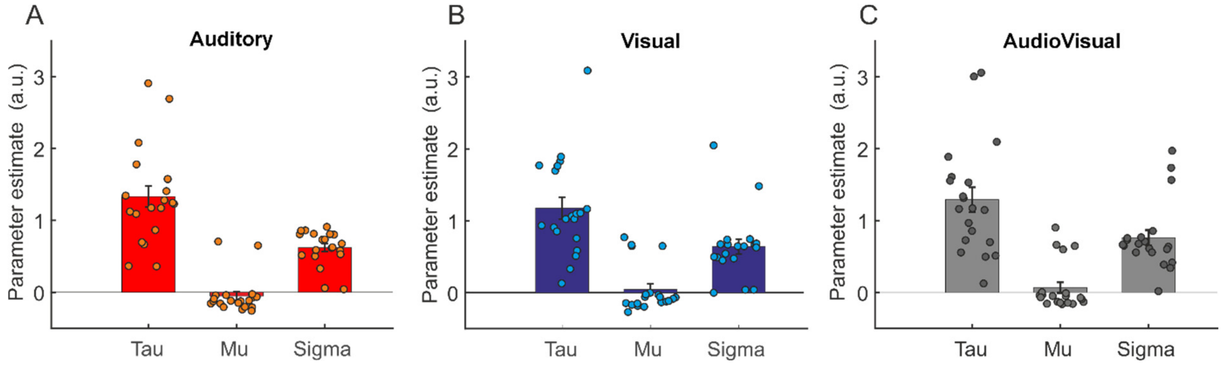

Figure S2: Mean of model-fit parameters across participants for the individual sensory modality conditions of the value task - (**A**) Auditory, (**B**), Visual, and (**C**) AudioVisual. Each dot represents the data of an individual participant. In order to test whether significant differences existed across sensory modality conditions for individual model-fit parameters, we performed a one-way repeated-measures ANOVA. We found no main effect of sensory modality on any of the three parameters: Tau ($F[2,38] = 1.72$, $p = 0.19$), Mu ($F[2,38] = 2.03$, $p = 0.15$), Sigma ($F[2,38] = 0.75$, $p = 0.48$). Therefore, the results shown in **Figure 2E** were pooled across all conditions.

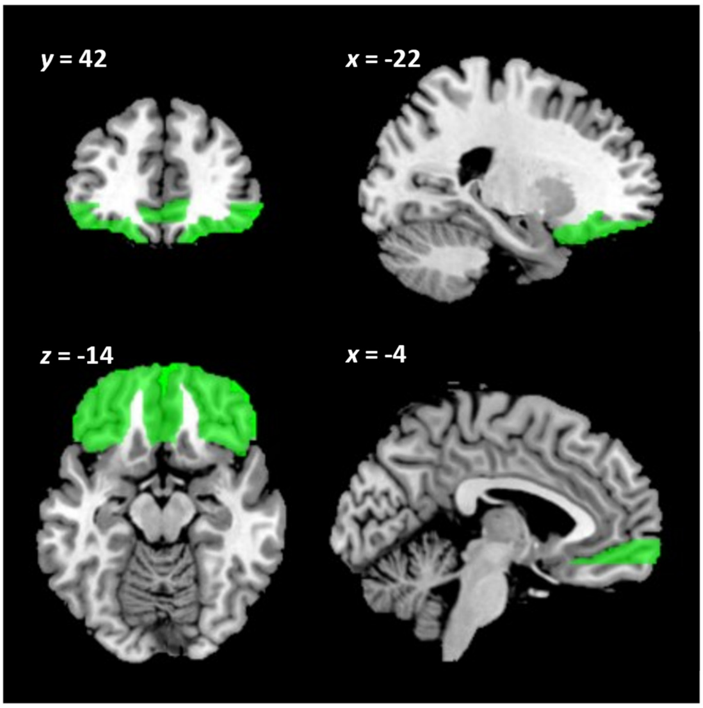

Figure S3. Anatomical definition of frontal valuation areas. The search volume used for multiple comparisons correction consisted of anatomical parcellations of the orbital surface of frontal gyrus as defined in automated anatomical labelling (AAL) atlas [1,2]. The search volume, comprised the anatomical parcellations of orbital surface in the following format, ROI name (abbreviation): Superior frontal gyrus - medial orbital (PFCventmed); Inferior orbital frontal gyrus (IFGorb); Medial orbital gyrus (OFCmed); Anterior orbital gyrus (OFCant); Posterior orbital gyrus (OFCpost); Lateral orbital gyrus (OFClat). For the detailed description of these areas see Table 2 in Rolls et al. 2015, 2020 [2,3].

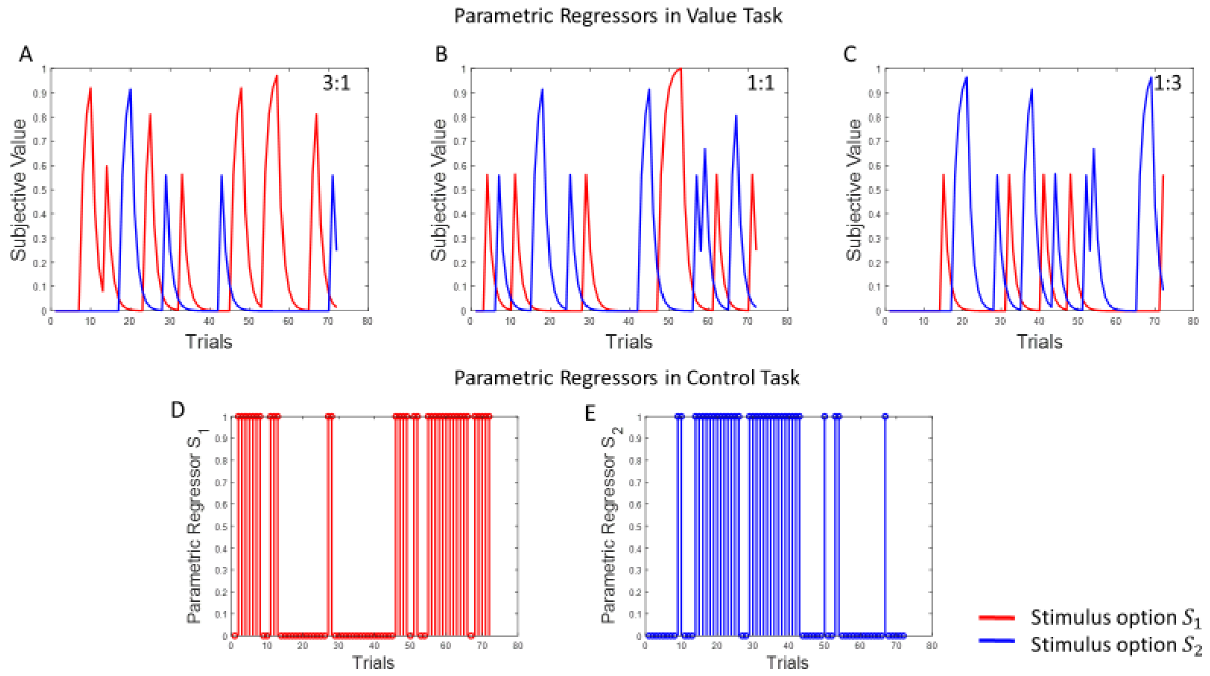

Figure S4. Illustration of the time course of parametric regressors in the value and control tasks. Parametric regressors used for the fMRI analysis are shown for a single participant. (**A-C**) For the value task, subjective values (SVs) of each option (S_1, S_2) are shown across trials in a block with reward ratios of 3:1, 1:1, 1:3, respectively. SVs were calculated based on the computational modeling of the behavioural data (see Methods in the main text). (**D-E**) For the control task, where instructions were passively followed across trials in a block, a weight of 0 or 1 was assigned to each option. The weights assigned to S_1 and S_2 in the control task were determined based on the schema shown in **Table S1**.

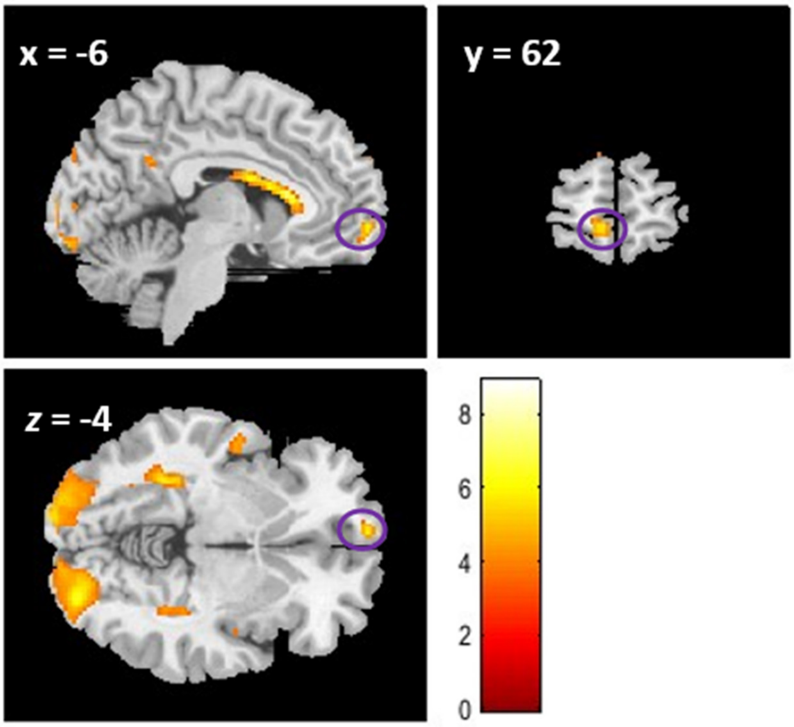

Figure S5. Activations in vmPFC in the control task. The activations correspond to the effect of parametric regressors across all modality conditions. The results are shown at the whole-brain uncorrected level of $P < 0.001$.

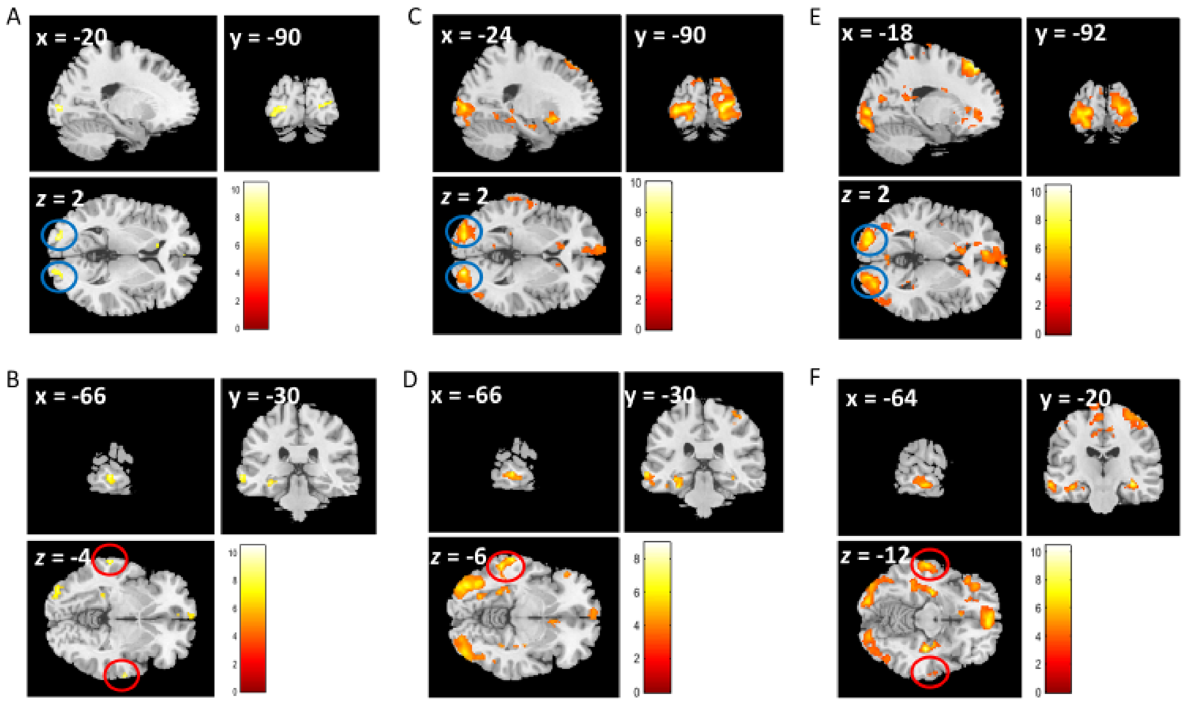

Figure S6. Stimulus value representations in sensory cortices: (**A**) Visual sensory cortex. (**B**) Auditory sensory cortex. All cluster activations shown in (A) and (B) were significant at whole-brain FWE corrected P < 0.05 and are estimated across all conditions (*AudAud, VisVis and AudVis*). (**C**) When the value modulations were inspected for individual conditions separately (whole-brain uncorrected level of P < 0.001), examination of the value parametric regressors in visual modality (i.e., contrast *intraVisSV* > 0) revealed activations in the visual sensory cortex (cluster peaks at (-24, -90, 2) and (18, -94, 8)) but no activation in the auditory sensory cortex were found. (**D**) Similarly, for the contrast *intraAudSV* > 0, we found activations in the auditory sensory cortex (cluster peak at (-66, -30, -6)) but no activation in the visual sensory cortex. This result indicates that each sensory cortex was maximally activated when the value of a stimulus from its specific modality was processed. However, for the latter contrast (*intraAudSV* > 0), we also found activations in higher visual areas (occipitotemporal cortex) with cluster peaks at (-36, -66, -8) and (42, -76, -12) that were distinct from activations found for the contrast *intraVisSV* > 0 which were in early visual areas in the occipital cortex (anatomical definitions are based on https://neurosynth.org/). (**E-F**) For the contrast *interAudVisSV* > 0, we found activations in both visual (cluster peaks at (-18, -92, 2) and (22, -90, 6)) and auditory (cluster peaks at (-64, -20, -12) and (66, -10, -4)) cortex, as in audio-visual condition trial-by-trial subjective values are updated individually for both auditory and visual options (whole-brain uncorrected level of P < 0.001). In all panels, crosshairs are placed at the left hemisphere cluster peak. The blue and red circles mark activations in the visual and auditory cortex, respectively.

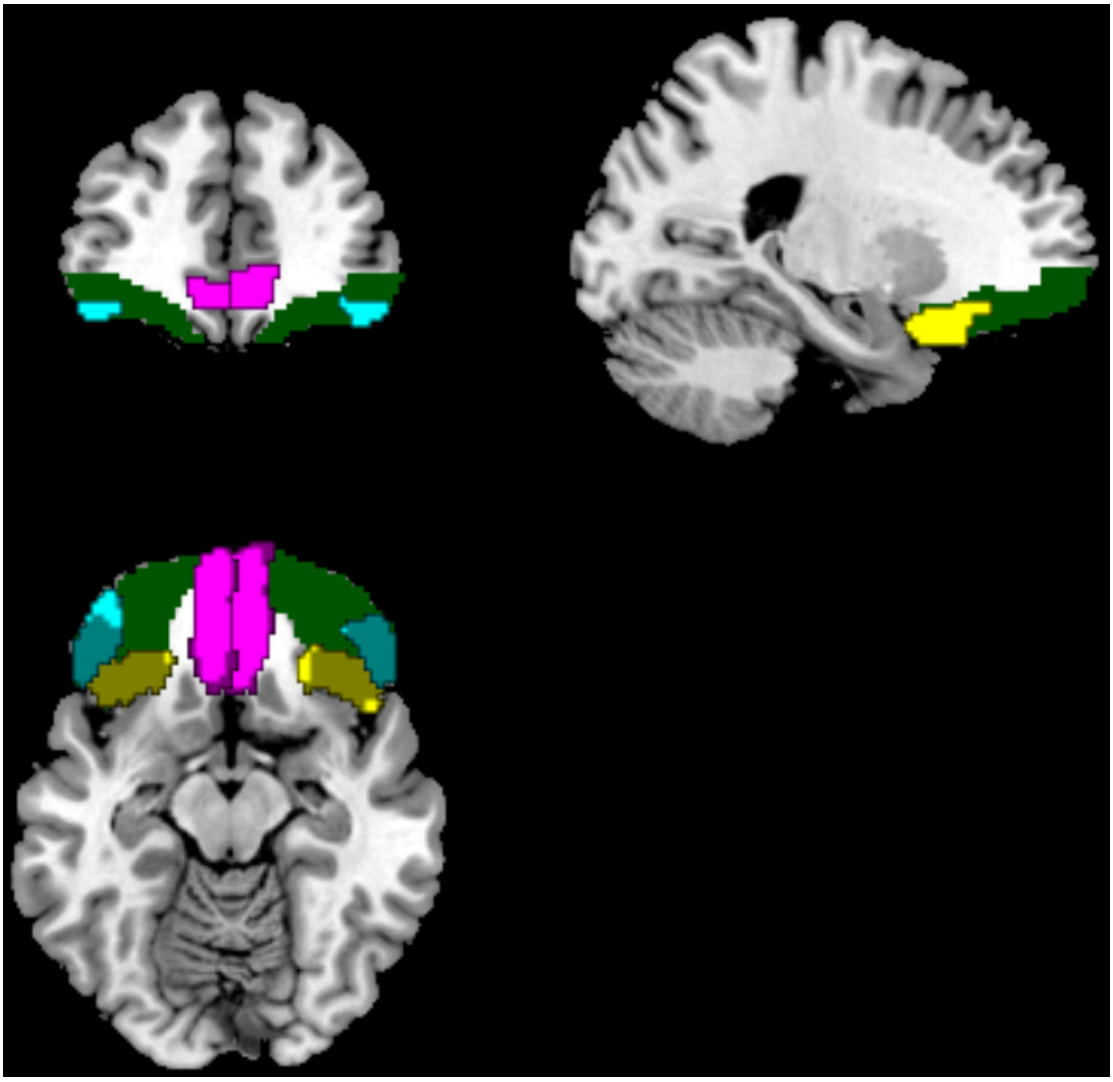

Figure S7. ROIs Used for the MVPA Analysis. We used three different anatomical ROIs from the AAL[2] atlas for our ROI-based MVPA analysis shown in Figure **4A-B**. The vmPFC (left and right) is shown in magenta, the posterior OFC (left and right) in yellow, and the lateral OFC (left and right) in cyan. The crosshair is at the MNI coordinate x = -22, y = 42, and z = -13. The whole frontal mask is shown in green for comparison, similar to **Figure S3**. The searchlight MVPA analysis in **Figure 4** was conducted within this mask.

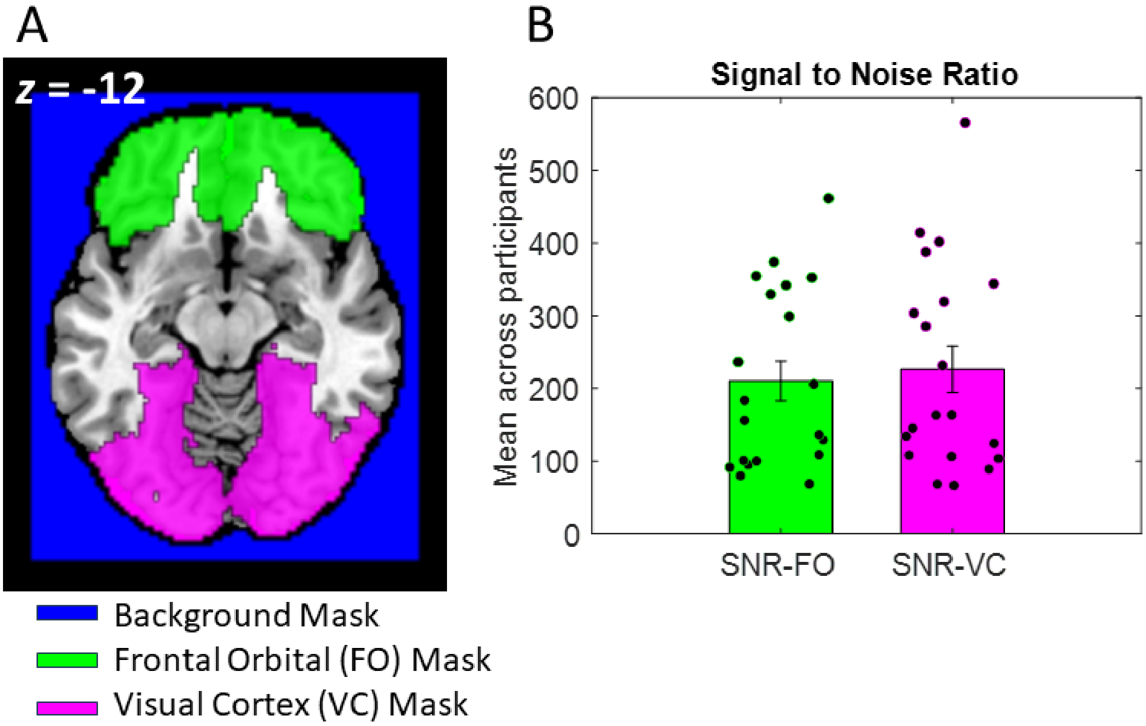

Figure S8. Signal to noise ratio comparison between frontal orbital region and visual cortex. (A) The background mask (or noise mask in blue), frontal mask (in green, as in **Figure S3**), and visual cortex (in magenta); cursor at z = -12. **(B)** The average SNR values from FO and VC regions across participants. Each data point corresponds to SNR in one individual participant.

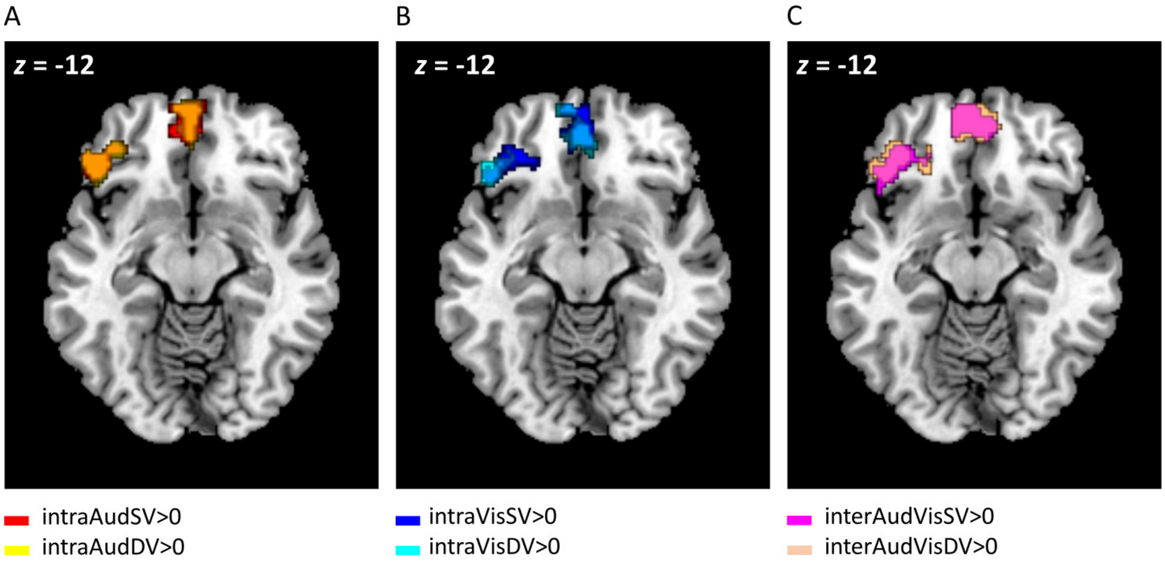

Figure S9. Stimulus Value Representations (SVRs) and representations of differential value (DVRs). Stimulus value representations (SVRs) identified based on the analysis shown in **Figure 3** are presented alongside the representations of the absolute differential value (absDV) for each condition of the value task: **(A)** intra-modal auditory, **(B)** intra-modal visual, and **(C)** inter-modal audiovisual.

Supplementary Text and Tables

Relationship between reward ratios in each modality and the probability of choice

We found a weak main effect of modality ($F[2,38] = 5.95$, $p = 0.024$) on choice ratios, indicating that choice ratios differed between modalities. This effect corresponded to a tendency of participants to choose the visual option more often than the auditory option in the audio-visual block even when they had the same reward ratio 1:1 as can be seen in **Figure 2A**, thereby creating a difference between choice ratios of intra- and inter-modal conditions. However, this difference only reached significance for the reward ratio 3:1 as in inter-modal trials as participants chose the auditory modality significantly less often than options in intra-modal trials (**Table S1**). Note that since LNP models fitted to the fMRI data were estimated for each condition separately, this bias (i.e., preference of visual over auditory stimuli in audiovisual blocks) did not have any impact on our reported results regarding the differences of value representations between modalities.

Table S1. Results of the post-hoc pairwise comparisons of choice ratios between different modalities.

Reward Ratio	Modality-1	Modality-2	Difference	pValue
**1:1**	Auditory	Visual	0.0417	0.9857
**1:1**	Auditory	AudioVisual	0.1069	0.0626
**1:1**	Visual	Auditory	-0.0417	0.9857
**1:1**	Visual	AudioVisual	0.0651	0.3084
**1:3**	Auditory	Visual	0.0548	0.3278
**1:3**	Auditory	AudioVisual	0.0701	0.2903
**1:3**	Visual	Auditory	-0.0548	0.3278
**1:3**	Visual	AudioVisual	0.0154	1.0
**3:1**	Auditory	Visual	0.0325	1.0
**3:1**	Auditory	AudioVisual	0.1424	0.0873
**3:1**	Visual	Auditory	-0.0325	1.0
**3:1**	Visual	AudioVisual	0.1098	0.0156*

* Indicates significance at $p<0.05$

Analysis of reaction times (RTs)

We conducted two analyses of RTs. Firstly, we carried out an analysis similar to the one done for choice ratios and shown in **Figure 2A-B**. The aim of the RT analysis was to test whether there was a systematic difference between the auditory and visual modalities in terms of their processing requirements, for instance the task difficulty. As shown in **Figure S1**, we found that intermodal responses were systematically slower than intra-modal responses $F[2,38] = 12.4$, $p < 0.001$, but importantly there was neither a significant effect of reward ratio nor interaction with modality (Fs<1 and ps>0.1), and RTs of auditory and visual intra-modal conditions were not significantly different (all ps>0.1). These results rule out the possibility that the observed segregations in neural representations of auditory and visual stimuli (**Figure 3A-E**) were due to their differences in other properties than their subjective values.

Secondly, similar to the analysis of the absolute differential value, we analysed the mean reaction time (RT) data for the two types of feedback in the value and the control tasks. A two-way repeated-measures ANOVA of RTs with task and feedback as factors revealed no significant main or interaction effects (p-values > 0.05). However, a trend was found for the main effect of task $F[2,38] = 4.76$, $p = 0.06$, reflecting faster responses in the control compared to the value task. Overall, the mean RT in the control task (787.5(±0.0613) ms), where participants had to simply follow instructions for decision-making was shorter than the mean RT in value task (824.4(±0.0635) ms).

Intuitively, a systematic decrease in the mean RTs of value task along with an increase in the absolute differential values (from no-reward to reward feedbacks), would indicate that participants take more time to reach a decision during difficult choice trials (when both options were perceived as having approximately equal values) in comparison to easy choice trials (when one option was clearly more valuable than the other). In the value task, mean (±s.e.m.) RTs decreased from 828.3(±0.0634) ms (no reward/blue feedbacks) to 819.7(±0.0638) ms (reward/yellow feedbacks). On the contrary, in the control task, mean RTs increased from 786.7(±0.0620) ms (keep/blue feedbacks) to 792.9(±0.0580) ms (switch/yellow feedbacks). Although insignificant, the latter effect implies an obvious fact that participants took less time when they had to keep their past choice in comparison to making a switch. Neither the main effect of feedback type on RTs nor their interaction with the task however reached significance, based on ANOVA (Fs<1, p>0.1).

Overview of model fits for individual sensory modalities

As shown in **Figure S2**, we did not find any evidence supporting that the fit parameters of the LNP model in the value task significantly differed across conditions. Below, we outline the fit parameters in each sensory modality separately (see also **Figure S2** alongside with the statistical comparisons done for each fit against zero). Overall, the time scale parameter τ was significantly greater than zero *in all conditions*, indicating that choices were in fact most impacted by recent rewards rather than distant rewards in the past (see also **Figure 2E**). Mean biasness μ in all conditions was not significantly different than zero, indicating that participants did not have a bias towards any particular option. The mean sensitivity σ was significantly greater than zero in all conditions, indicating that participants were aware of the value difference between options and had indeed adopted an optimal balance between exploration and exploitation (σ = 0 and $\sigma \gg 1$ for extreme exploitative and explorative tendencies, respectively).

Table S2: Mean of model-fit parameters across participants and one-sample t-test statistics – for individual conditions (Aud, Vis, AudVis) of the value task

**Modality**	**Tau – Mean(±s.e.m.) (t-test statistics)**	**Mu – Mean(±s.e.m.) (t-test statistics)**	**Sigma – Mean(±s.e.m.) (t-test statistics)**
**Auditory**	1.33(±0.15s.e.m.) (t[19] = 8.99, $p < 0.05$)	-0.05(±0.06s.e.m.) (t[19] = -0.89, $p = 0.39$)	0.62(±0.05s.e.m.) (t[19] = 11.33, $p < 0.05$)
**Visual**	1.18(±0.15s.e.m.) (t[19] = 7.80, $p < 0.05$)	0.05(±0.07s.e.m.) (t[19] = 0.64, $p = 0.53$)	0.64(±0.10s.e.m.) (t[19] = 6.29, $p < 0.05$)
**Intermodal**	1.29(±0.17s.e.m.) (t[19] = 7.44, $p < 0.05$)	0.07(±0.07s.e.m.) (t[19] = 0.93, $p = 0.37$)	0.76(±0.11s.e.m.) (t[19] = 7.22, $p < 0.05$)

Overview of all regressor types in univariate GLMs

Table S3. Regressors-of-interest included in the univariate analysis				
**Order**	**Regressor Type**	**Condition**	**Task**	**Timing**
**1**	Trial Identity (unmodulated)	AudAud	Value	Onset at stimuli interval
**2**	Parametric (value-modulated)	AudAud (LP)		
**3**	Parametric (value-modulated)	AudAud (HP)		
**4**	Trial Identity (unmodulated)	VisVis		
**5**	Parametric (value-modulated)	VisVis (R)		
**6**	Parametric (value-modulated)	VisVis (G)		
**7**	Trial Identity (unmodulated)	AudVis		
**8**	Parametric (value-modulated)	AudVis (A)		
**9**	Parametric (value-modulated)	AudVis (V)		
**10**	Trial Identity (unmodulated)	AudAud	Control	Onset at stimuli interval
**11**	Parametric (instruction followed or not)	AudAud (LP)		
**12**	Parametric (instruction followed or not)	AudAud (HP)		
**13**	Trial Identity (unmodulated)	VisVis		
**14**	Parametric (instruction followed or not)	VisVis (R)		
**15**	Parametric (instruction followed or not)	VisVis (G)		
**16**	Trial Identity (unmodulated)	AudVis		
**17**	Parametric (instruction followed or not)	AudVis (A)		
**18**	Parametric (instruction followed or not)	AudVis (V)		
19	Response (unmodulated)	Common	Common	Time of response
20	Feedback (unmodulated)	Common	Common	Onset at feedback interval
21-35	Nuisance regressors			

Regressor **1-18** marked in **bold** are of event-related type and are different across the value and control tasks (1-9 for value and 10-18 for the control task). Regressors marked as common were event-related regressors appearing at the time of Response or Feedback in all conditions of both tasks. Regressors 21-35, were nuisance regressors, resulting in a total of 35 regressors.

Definition of parametric regressors for the control task

Similar to the value task, two regressors were modelled at the onset of stimuli options in the control task: one unmodulated regressor representing the modality-wise trial identity and two parametrically modulated regressors for each of the two choice options (S_1 and S_2). To define the parametric regressors, we assigned a weight of either 1 or 0 to each option according to the schema shown in **Table S4**.

**Table S4. Definition of parametric regressors for the control task***		
**Choice on trial *t*-1**	**Instruction from trial *t*-1**	**Weight assigned on trial *t***
S_1	Not followed	S_1 - 0, S_2 - 0
S_2	Not followed	S_1 - 0, S_2 - 0
S_1	Followed; Instruction was to keep	S_1 - 1, S_2 - 0
S_1	Followed; Instruction was to switch	S_1 - 0, S_2 - 1
S_2	Followed; Instruction was to keep	S_1- 0, S_2 - 1
S_2	Followed; Instruction was to switch	S_1 - 1, S_2 - 0

*When instruction from the previous trial (*t*-1) was not followed, a weight of 0 was assigned to both options S_1 and S_2. When the instruction from the previous trial *t*-1 was correctly followed, the option that corresponded to the correct instructed choice in trial *t* received a weight of 1 and the other option received a weight of 0.

Stimulus Value Representations (SVRs) measured based on individual parametric regressors in the value task

The univariate results reported in the main text and shown in **Figure 3** and **Table 1** were based on contrasting both parametric value regressors from a condition, say *lpSV* and *hpSV* in intra-modal auditory condition (corresponding to low-pitch and high-pitch auditory stimuli), together against the baseline (i.e., *lpSV* + *hpSV* >0) to determine the overall subjective value representations of a particular stimulus modality condition. An alternative analysis is to contrast individual value regressors against baseline (i.e. either *lpSV* >0 or *hpSV* >0). This allows us to test whether representations of individual stimulus options within a certain modality condition (e.g. red and green in visual, intra-modal condition and high or low pitch stimuli in auditory intra-modal conditions) have overlapping representations.

Therefore, we undertook the following analyses. In intra-modal auditory condition (*AudAud*) of the value task, we examined the parametric value regressors separately for the low-pitch and high-pitch tones (referred to as *intraAud_lpSV* > 0 and *intraAud_hpSV* > 0 contrasts, respectively; for details see Methods and **Table 1** in the main text and **Table S5**). Both aforementioned auditory contrasts revealed activations in vmPFC and left lateral OFC (latOFC) (see **Table S5**), which were adjacent to the clusters found in our main analysis (reported in Table 1 corresponding to contrast *intraAudSV* > 0). Similarly, in intra-modal visual condition (*VisVis*) of the value task, we examined the parametric value regressors separately for the red and green checkerboards (referred to as *intraVis_rSV* > 0 and *intraVis_gSV* > 0 contrasts, respectively; see **Table S5**). Both these visual contrasts revealed activations in vmPFC and left posterior OFC (postOFC) (see **Table S5**), close to the cluster seen in our main analysis for intra-modal visual SVs (reported in **Table 1** corresponding to contrast *intraVisSV* > 0). Thus, as expected, in both intra-modal conditions, the representations from the two value regressors were found to be overlapping both in vmPFC and OFC. Moreover, the value representations from the two sensory modalities were found to be overlapping in vmPFC, but not in OFC.

Furthermore, we performed a similar analysis for inter-modal condition (*AudVis*) of the value task, where we examined the parametric value regressors separately for the auditory and visual stimuli (referred to as *interAudVis_aSV* > 0 and *interAudvis_vSV* > 0 contrasts, respectively; see **Table S5**). These contrasts also revealed significant activations in vmPFC and left lateral and posterior OFC, which were close to their respective intra-modal counterparts (d< 8mm). The peaks reported in **Table S5** for inter-modal conditions, were then used to extract ROIs

shown in **Figure 3G**. In these ROIs, for instance in the visual ROI defined based on the *interAudVis_vSV >0* contrast, we measured the effect sizes for all other stimuli, for instance *intraVis_rSV > 0* and *intraVis_gSV > 0* in intra-modal visual condition and *intraAud_hpSV> 0* and *intraAud_lpSV > 0* in intra-modal auditory condition. This way, we could assess whether the modality-specificity generalizes across independent datasets when the definition of ROIs and test of effect sizes is done independently (**Figure 3G**).

We note that contrasting individual regressors against the baseline had lower power, as some activations in **Table S5** did not reach significance even at a more liberal threshold (P = 0.005). This lower power is likely because the overall variance was divided between the two parametric value regressors, reducing the effectiveness of BOLD response estimation (lower signal-to-noise ratio). However, the results largely support the findings of our main analyses in Table 1 and **Figure 3**.

Table S5. Stimulus value representations in vmPFC and OFC for individual value-modulated regressors contrasted against baseline

**Contrast**	**Region**	**X**	**Y**	**Z**	**t(19)**	**k**	**SVFWE corr. P**
intraAud_lpSV> 0	vmPFC_L	0	62	-4	3.66	322	**0.007**
	latOFC_L	-48	32	-8	3.92	100	0.166
intraAud_hpSV> 0	vmPFC_L	-6	64	-2	4.12	91	0.155
	latOFC_L	-48	28	-14	4.33	176	**0.031**
intraVis_rSV > 0	vmPFC_L	4	58	-10	3.24	39	0.464
	postOFC_L	-32	28	-16	4.21	105	0.114
intraVis_gSV > 0	vmPFC_L	-4	62	-2	3.99	282	**0.003**
	antOFC_L<br>*(postOFC_L)*	-34<br>(-30	36<br>26	-16<br>-18)	4.07<br>(3.73)	170	**0.024**
interAudVis_aSV >0	vmPFC	-2	52	-8	3.61	310	**0.005**
	latOFC_L	-48	32	-6	6.04	558	**P < 0.001**
interAudVis_vSV >0	vmPFC_L	-4	58	-10	6.81	792	**P < 0.001**
	antOFC_L<br>*(postOFC_L)*	-34<br>(-36	36<br>24	-12<br>-18)	7.89<br>(3.52)	334	**0.003**

MNI coordinates (x, y, z) and T value corresponds to the local maxima peak of the cluster activations at SVFWE corrected $P < 0.05$ (cluster labels are from AAL atlas [54]). Statistical maps were assessed for cluster-wise significance using a cluster-defining threshold of $t(19) = 2.86$, $P = 0.005$; and using small volume corrected threshold of P-value (reported in right-most column; referred to as a small volume family-wise-error (SVFWE) correction) within the frontal search volume.
Activations shown in *italic* correspond to conditions in which more than one peak was detected in a larger cluster.

IntraAud_lpSV>0: contrast capturing responses elicited by changes in subjective value of low pitch auditory stimulus (*lpSV*) in intra-modal auditory condition.
IntraAud_hpSV>0: contrast capturing responses elicited by changes in subjective value of high pitch auditory stimulus (*hpSV*) in intra-modal auditory condition.
IntraVis_rSV>0: contrast capturing responses elicited by changes in subjective value of red-coloured checkerboard visual stimulus (*rSV*) in intra-modal visual condition.
IntraVis_gSV>0: contrast capturing responses elicited by changes in subjective value of green-coloured checkerboard visual stimulus (*gSV*) in intra-modal visual condition.
InterAudVis_aSV>0: contrast capturing responses elicited by changes in subjective value of auditory stimulus (*aSV*) in inter-modal audio-visual condition.
InterAudvis_vSV>0: contrast capturing responses elicited by changes in subjective value of visual stimulus (*vSV*) in inter-modal audio-visual condition.

Comparison of the value and the control task

In addition to the value task, we also inspected the control task using the same contrasts that were used to detect modality-specific and modality-general representations shown in **Figure 3**. Interestingly, we found that in the control task there were activations (**Figure S5** and **Table S6**) in vmPFC that overlapped with modality-general representations that were found in the value task. This observation indicates that a task with comparable choice structure, but no valuation requirement also involves vmPFC, underscoring the role of this region as a general comparison and choice computation region. No activations were found at the OFC with these contrasts at the reported threshold.

Table S6. Univariate contrasts inspected in the control task

Contrast	Region	*x*	*y*	*z*	*t*(19)	*k*	*SVFWE corr P*
Control_intraAud > 0	vmPFC	-8	62	-4	4.49	11	0.300
Control_intraVis > 0	vmPFC	-6	64	-6	4.37	12	0.287
Control_interAudVis > 0	vmPFC	-4	56	-12	2.87	8	0.966

MNI coordinates (x, y, z) and T value corresponds to the local maxima peak of the cluster activations (cluster labels are from AAL atlas). Statistical maps were assessed for cluster-wise significance using a cluster-defining threshold of $t(19) = 3.58$, $P = 0.001$; and using small volume correction within the frontal search volume but cluster-level SVFWE corrected P-values were above threshold P-value 0.005 (as shown in last column of the table).

Description of contrasts:

Control_intraAud>0: contrast capturing the overall responses to the parametric regressors corresponding to both auditory stimuli in intra-modal (*AudAud*) condition of control task. Note that in this case, the contrast vector contains 1 for the two parametric regressors of interest, here both auditory stimuli, and 0 otherwise.

Control_intraVis>0: contrast capturing the overall responses to the parametric regressors corresponding to both visual stimuli in intra-modal (*VisVis*) condition of control task. Note that in this case, the contrast vector contains 1 for the two parametric regressors of interest, here both visual stimuli, and 0 otherwise.

Control_interAudVis>0: contrast capturing the overall responses to the parametric regressors corresponding to auditory and visual stimuli in inter-modal (*AudVis*) condition of control task. Note that in this case, the contrast vector contains 1 for the two parametric regressors of interest, here auditory and visual stimuli, and 0 otherwise.

Effective Connectivity Analysis

The model space shown in **Figure 5** and **Figure 6** was developed over a base model comprising driving inputs and intrinsic connections, which did not vary with the experimental conditions. The rest of the models differed from each other over modulatory connections, which depended on the experimental conditions **(Figure 5A)**. In the base model, intrinsic connections were defined between every pair of nodes in the network and as self-connections. In **Table S7** and **S8**, we show the estimated strength of the intrinsic connectivity (**Table S7**) and driving inputs (**Table S8**) of the winning model.

Table S7. Intrinsic connectivity parameters in the network including self-connectivity

To\From	vmPFC	audOFC	visOFC	audSen	visSen
**vmPFC**	-1.4645	-0.0433	0.2394	-0.0680	-0.0310
**audOFC**	-0.1555	-1.1751	0.2242	-0.0215	0.0809
**visOFC**	0.0250	-0.0477	-0.8745	0.0015	0.0514
**audSen**	0.0491	-0.0508	-0.0251	-0.9244	0.0671
**visSen**	-0.0213	0.0334	-0.0159	0.0005	-1.1425

All parameters are significant at posterior probability of P > 0.99

Table S8. Driving input influence parameters on sensory ROIs of the network

ROIs\Driving Input*	intraAud	intraVis	interAud	interVis
**audSen**	0.0042	0	0.0226	0.0224
**visSen**	0	-0.0025	-0.0035	0.0240

All parameters are significant at posterior probability of P > 0.99
*For trial-types of different conditions of the value task as described in the methods section on EC

Quality of signal in the frontal area

To account for the signal dropout in the frontal regions, the following steps were taken during the data acquisition: 1) We used a data acquisition protocol that minimizes signal dropout in the regions [4]: for this a PA phase encoding direction was used in combination with a rather high in-plane spatial resolution and rather thin slices (2x2x2mm) for our EPIs, and 2) we took special steps during the data acquisition: after every fMRI run we acquired a field map (instead of just one field map per session) to have an as close as possible match between fMRI and field map to correct image distortions during the data analysis.

Furthermore, in order to quantify signal quality and compare frontal regions-of-interest (Frontal Orbital (FO) mask in **Figure S3**) with regions which are less susceptible to dropout such as visual cortex, we compared the signal to noise ratio (SNR) across the FO region and visual cortex (VC, shown in **Figure S8**). SNR is a metric for assessing the acquisition quality of fMRI images, where the mean fMRI signal activity is divided by the standard deviation of the background noise signal [5]. The background noise signal is measured from the voxels of the images where no anatomical matter exists [6]. In order to extract the background mask, shown in **Figure S8**, we employed tissue probability maps in MNI-space available in SPM12 and masked the anatomical matter regions as zeros and background brain voxels as one. Specifically, for each participant we evaluated the SNR separately from FO and VC, (SNR_FO: 210.29 ± 27.41 (mean ± s.e.m.); SNR_VC: 226.28 ± 31.84 (mean ± s.e.m.), shown in **Figure S8**), and we found that there was no significant difference in the two ratios (paired t-test $t[19] = -1.9491$, $p = 0.0662$), indicating that signal coverage in key frontal regions was sufficiently good and signal dropout was not a problem.

Absolute differential value representations in frontal cortex – univariate analysis

In a binary choice situation such as in the value task we employed, the ultimate choice is guided by the differential value (DV) between the two options, leading to a choice of an item that is believed to have a higher value. In the main analysis reported in the main text, we however focused on the subjective value (SV) of each of the two options, since the valuation process prior to the choice was the focus of our study. To test the degree to which these two types of representations overlap we also examined a GLM that included a parametric regressor for the absolute differential value. This GLM was similar to our main univariate analysis GLM except that in the value task, for each condition the two subjective value parametric regressors

were replaced with one absolute differential value (absDV) regressor, which modelled the absolute difference between the trial-by-trial modulations in participants' beliefs regarding the value of each stimulus option. This GLM has an additional advantage that due to inclusion of only one parametric regressor per trial, the collinearity between regressors was reduced compared to a GLM that includes two parametric regressors for each choice option. The absolute DV representations of each modality condition were then examined in the frontal cortex (**Figure S3**) based on a group-level random-effects analysis on the contrast images obtained from all participants.

In intra-modal conditions (*AudAud* and *VisVis*), we examined the absolute differential value regressors separately for the auditory or visual sensory modality (referred to as *intraAudDV* > 0 and *intraVisDV* > 0 contrasts, respectively; for details see footnote of **Table S9**). Further, we examined the absDV regressor for inter-modal condition (*AudVis*) (denoted by *interAudVis > 0*). The results from all these three contrasts strongly supported the results from our main univariate analysis, indicating the robustness of our findings (**Figure S9**).

Table S9. Absolute differential value representations in vmPFC and OFC for various univariate contrasts

Contrast	Region (SVFWE)	*X*	*Y*	*Z*	*t*(19)	*k*
intraAudDV > 0	vmPFC_L	-6	66	-2	4.81	116
	latOFC_L	-48	32	-8	5.21	104
intraVisDV > 0	vmPFC_L	-10	58	-14	4.90	196
	postOFC_L	-30	26	-18	5.63	130
interAudVisDV > 0	vmPFC	2	55	-10	7.11	619
	antOFC_L	-40	36	-14	5.73	345

MNI coordinates (x, y, z) and T value corresponds to the local maxima peak of the cluster activations at SVFWE corrected $P < 0.005$ (cluster labels are from AAL atlas [61]). Statistical maps were assessed for cluster-wise significance using a cluster-defining threshold of $t(19) = 3.58$, $P = 0.001$; and using small volume corrected threshold of $P < 0.005$ (referred to as a small volume family-wise-error (SVFWE) correction) within the frontal search volume.

Description of contrasts:

IntraAudDV>0: contrast capturing responses elicited by changes in absolute differential value (DV) when choice options consisted of two auditory stimuli (*AudAud*). This contrast was calculated as *intraAudDV*>0, corresponding to the responses to the absolute difference between the trial-by-trial parametric values of low and high pitch auditory stimuli *(lpSV* and *hpSV*, respectively).

IntraVisDV>0: contrast capturing responses elicited by changes in absolute differential value (DV) when choice options consisted of two visual stimuli (*VisVis*). This contrast was calculated as *intraVisDV*>0, corresponding to the responses to the absolute difference between the trial-by-trial parametric values of the green and red colours of visual stimuli (*gSV* and *rSV*, respectively).

InterAudVisDV>0: contrast capturing responses elicited by changes in absolute differential value (DV) when choice options consisted of one auditory and one visual stimulus (*AudVis*). This contrast was calculated as *interAudVisSV>0*, corresponding to the responses to the absolute difference between the trial-by-trial parametric values of the auditory and visual stimuli (*aSV* and *vSV*, respectively).

Citations

1. Rolls, E. T., Joliot, M. & Tzourio-Mazoyer, N. Implementation of a new parcellation of the orbitofrontal cortex in the automated anatomical labeling atlas. *Neuroimage* **122**, 1–5 (2015).
2. Rolls, E. T., Huang, C. C., Lin, C. P., Feng, J. & Joliot, M. Automated anatomical labelling atlas 3. *Neuroimage* **206**, 116189 (2020).
3. Rolls, E. T., Joliot, M. & Tzourio-Mazoyer, N. Implementation of a new parcellation of the orbitofrontal cortex in the automated anatomical labeling atlas. *Neuroimage* **122**, 1–5 (2015).
4. Weiskopf, N., Hutton, C., Josephs, O., Turner, R. & Deichmann, R. Optimized EPI for fMRI studies of the orbitofrontal cortex: compensation of susceptibility-induced gradients in the readout direction. *MAGMA* **20**, 39 (2007).
5. Baijot, J. *et al.* Signal quality as Achilles' heel of graph theory in functional magnetic resonance imaging in multiple sclerosis. *Scientific Reports 2021 11:1* **11**, 1–9 (2021).
6. Welvaert, M. & Rosseel, Y. On the Definition of Signal-To-Noise Ratio and Contrast-To-Noise Ratio for fMRI Data. *PLoS One* **8**, e77089 (2013).
